# Self-Construal Priming Modulates Ensemble Perception of Multiple-Face Identities

**DOI:** 10.3389/fpsyg.2019.01096

**Published:** 2019-05-16

**Authors:** Shenli Peng, Ling Zhang, Runzhou Xu, Chang Hong Liu, Wenfeng Chen, Ping Hu

**Affiliations:** ^1^ Department of Psychology, Renmin University of China, Beijing, China; ^2^ Department of Psychology, Bournemouth University, Poole, United Kingdom

**Keywords:** interdependent self-construal, independent self-construal, ensemble perception, high-level feature, low-level feature

## Abstract

This study explored the modulatory role of independent/interdependent self-construal on ensemble perception. Two experiments were conducted to study the effect of self-construal on ensemble coding of multiple-face identities (Experiment 1) and dot size (Experiment 2) separately. Before the implicit ensemble perception task, participants in both experiments were either primed with independent or interdependent self-construal *via* a well-validated pronoun circle task, in which they were exposed to either singular (“*I*,” “*me*,” and “*my*”) or plural (“*We*,” “*us*,” and “*our*”) pronouns in essays. The results showed that interdependent self-construal (vs. independent self-construal) featured as global processing and emphasizing interconnectedness with others enhanced the ensemble coding of high-level features (e.g., identity in Experiment 1) but not of low-level features (e.g., size in Experiment 2). To the best of our knowledge, this study was the first to investigate the role of self-construal on ensemble representations. In sum, the results of the current study supported the domain-specific mechanism of ensemble perception on one hand, and extended the effect of self-construal on single face recognition to multiple face recognition on the other hand.

## Instruction

People encounter many redundant objects in everyday life, such as clumps of bushes, rows of shelves, bunches of bananas, and groups of people. How do people perceive great volumes of information with known limited cognitive resources (Chong and Treisman, 2003; Luck and Vogel, 2013; Cohen et al., 2016)? To figure this out, previous studies have endorsed the existence of a fast visual-averaging mechanism in humans, a phenomenon termed ensemble perception (Ariely, 2001; Haberman and Whitney, 2007, 2009; see Alvarez, 2011, and Whitney and Yamanashi Leib, 2018, for reviews). Ensemble perception refers to our visual ability to rapidly extract or compute summary statistical information from a set of homogeneous objects (Whitney and Yamanashi Leib, 2018), which is evidenced in many feature domains, including size (Ariely, 2001), orientation (Parkes et al., 2001), hue (Maule and Franklin, 2015), gender (Haberman and Whitney, 2007), identity (de Fockert and Wolfenstein, 2009), and emotion (Haberman and Whitney, 2009). For instance, people are adept at computing the mean emotion of a group of emotional expressions in a remarkably short time, even without precise knowledge of individual expressions (Haberman and Whitney, 2009). Ensemble perception is of evolutionary significance for its role in facilitating outlier detection (Haberman and Whitney, 2010), attention orienting (Alvarez, 2011; Haberman and Whitney, 2012), and information compression in visual working memory (Brady and Alvarez, 2011).

Given that evidence shows ensemble perception occurs in a remarkably short time (e.g., Haberman and Whitney, 2009), many researchers have assumed that ensemble properties are extracted by pooling information from all or most items in a set, which is a global process requiring distributed attention (Chong and Treisman, 2005; Alvarez, 2011; Srinivasan, 2017). For example, Chong and Treisman (2005) tested whether distributed attention was conducive to ensemble perception by letting participants accomplish a mean size extraction task combined with a concurrent task requiring either global or local attention. The results indicated that participants did better in mean size extraction tasks, when they were combined with tasks requiring global attention rather than local attention, which suggests that representing ensemble properties requires global attention to the set information.

Evidence concerning the global-processing nature of ensemble perception could also be extracted from previous empirical studies. In a recent study (Im et al., 2017), Korean and American participants were enrolled in a comparison of an avoidance task based on the visual averaging of emotions of facial crowds. Specifically, participants from the two countries were instructed to make an avoidance choice in light of the average emotion of two groups of emotional faces on both sides of the display. The results showed that the Korean participants were more adept at the averaging task, which the authors suggested was primarily due to the habitual global-processing style of the Korean participants.

Consistent with Im et al., in our recent study (Peng et al., under review), we adopted two studies to confirm the facilitating effect of the global-processing style on ensemble perception. In Study 1, we compared Chinese and British participants’ performance in representing the average identity of multiple faces; the results indicated that Chinese participants with a habitual global-processing orientation were more likely to erroneously endorse the average identity as a member of the preceding set than were the British participants with a chronic local processing orientation. In Study 2, we temporarily activated (primed) participants from one culture (China) with either a global or local processing orientation and asked them to perform the ensemble coding task, as in Study 1; the results demonstrated that participants exposed to a global-processing orientation priming displayed an ensemble coding superiority over their counterparts who were exposed to a local processing orientation. Together, these studies indicated that ensemble perception might draw on a global-processing style. However, it remains in dispute whether the evidence suggests that a focused attention mode is also or alternatively responsible for the representation of statistic summaries (e.g., de Fockert and Marchant, 2008; Myczek and Simons, 2008; Marchant et al., 2013). Thus, further studies have been needed to tackle this issue. This led to the first aim of this study, which was to explore the effect of global vs. local processing, manipulated by interdependent and independent self-construal priming, respectively, on ensemble perception.

Self-construal has mostly been used by social psychologists to explore its effect on cognition and emotion processing. Variations in chronic self-construals among different cultures were evidenced to affect global and local processing (Masuda and Nisbett, 2001; Nisbett et al., 2001; Nisbett and Miyamoto, 2005; Lao et al., 2013; Hess et al., 2016). For example, McKone et al. (2010) compared global-local processing differences between East Asians and Caucasian Westerners through a Navon letter identification task, in which participants had to respond to target letters that could be (randomly) either a small local letter or a large global letter; the results showed that East Asians with chronic interdependent self-construal displayed a global-processing superiority relative to Caucasians with chronic independent self-construal. Furthermore, this global advantage extended to second-generation immigrant families. However, since self-construals could also be momentarily changed by priming techniques (e.g., pronoun circle task), researchers have also explored the effect of self-construal on global and local processing. Lin and Han (2009) explored the effect of Eastern interdependent self-construal priming vs. Western independent self-construal on the cognitive processing style. Their studies demonstrated that interdependent rather independent self-construal priming enlarged the scope of visual attention, leading to a more global-processing style. In summary, people with an interdependent self-construal, whether chronic or temporarily activated, will more likely perceive the world in a global-processing style, while people with an independent self-construal will tend to process objects in a more local way. Thus, one may speculate that an interdependent self-construal would facilitate ensemble perception.

Although the ubiquitous nature of ensemble perception is well-confirmed (Alvarez, 2011; Whitney and Yamanashi Leib, 2018), Haberman et al. (2015) suggested its process is a domain-specific mechanism. In a series of experiments employing high-level (identity, emotion) and low-level (orientation, color) features, Haberman and his colleagues enrolled participants into two tasks: an individual member-identification task and an ensemble coding task. During the individual task, participants were cued to observe one target Gabor or face of a total of four items (e.g., Gabors or face identities) and instructed to adjust the test item to match the cued individual while ignoring other distracters. During the ensemble task, participants were cued to observe four items and asked to adjust the test item to match the average representation of the prior set. The authors uncovered that average performances of low-level and high-level features were independent of each other, while the within-feature-domain ensemble representations were highly correlated with each other. For example, ensemble representations of face identity and emotional expression were correlated with each other, which was the case among ensemble representations of orientation and color. Haberman et al. indicted there was a dissociation between ensemble perceptions of the high- and low-level features domain, which has been supported by many studies (Emmanouil and Treisman, 2008; Albrecht et al., 2012; Hubert-Wallander and Boynton, 2015). Thus, the second aim of this study was to explore whether the effect of self-construal priming on ensemble coding of high-level (identity) differed or not from that of low-level (size) features, as suggested by the domain-specific view.

To achieve this, two experiments were conducted, with Experiment 1 exploring the causal role of self-construal in ensemble coding of multiple face identities and Experiment 2 exploring the role of self-construal in multicircle size averaging. Following prior research, we adopted the self-construal priming technique. Previous studies indicated that although self-construal is formed by culture, it can be temporarily adjusted by the pronoun-circle task, which requires participants to search for independent or interdependent pronouns (e.g., “I” or “we”) in short essays (Gardner et al., 1999; Heine, 2001). In China’s context, the pronoun circle task has been well-validated and popularly used in self-construal priming research (Sui and Han, 2007; Zhang et al., 2017). On arrival at the laboratory, participants in both experiments were randomly assigned into either the independent or interdependent self-construal priming group. After that, participants had to accomplish the implicit ensemble coding task. The task we adopted in this study was adapted from de Fockert and Wolfenstein (2009), in which participants were first presented with a set of different items, and then, when shown another display, they had to decide whether a test item had been present or not in the preceding set. The test stimulus consisted of four conditions: (1) an average representation of the preceding set (match average); (2) an average representation of another stimulus set (nonmatch average); (3) an exemplar of the preceding set (match exemplar); and (4) an exemplar of another set (nonmatch exemplar). Note that participants were unaware of these four conditions of the test item. Proportions of “present” responses of each condition were employed as an index of ensemble perception; for example, de Fockert and Wolfenstein (2009) found that participants in their study reported a comparable or even higher proportion of “present” responses to match average faces than to match exemplar faces, indicating that participants implicitly extracted average faces from the preceding face set. To acquire more nuanced evidence for the ensemble perception, an unbiased index, an endorsement score was recently proposed and confirmed by Rhodes and colleagues (Rhodes et al., 2015, 2017). By subtracting the proportions of “present” responses of nonmatch conditions from those of match conditions for both the set average and exemplar, endorsement scores could exclude the possible effects of image features, such as texture. However, there no research has examined ensemble representations of low-level features (e.g., size) *via* the implicit ensemble coding task.

For Experiment 1, we hypothesized an interdependent self-construal priming (vs. independent self-construal priming) and elevated the visual averaging performance of multiple face identities, since the interdependent self-construal priming could shift participants to a more global-processing style, which was conducive to the ensemble perception. For Experiment 2, we hypothesized a different results pattern than for Experiment 1, according to the domain-specific mechanism of ensemble perception; however, the hypothetical results were unspecified since no research has measured ensemble coding of size with an implicit ensemble perception task.

## Experiment 1

### Participants

A total of *n* = 52 (14 males, age = 19.62 ± 1.97 years old) right-handed college students from Renmin University of China (RUC) were recruited to take part in this study, with monetary compensation. The required sample size was calculated using the free software G Power (Faul et al., 2009) for 80% power, which has been commonly used in past research (e.g., Tressoldi, 2012). All participants had normal or corrected-to-normal vision. Participants were randomly assigned into two groups: (1) one independent self-construal priming group (*n* = 27, 9 males, age = 19.56 ± 2.36 years old); and (2) one interdependent self-construal priming group (*n* = 25, 5 males, age = 19.68 ± 1.49 years old). This study was approved by the Institutional Review Board of Department of Psychology, RUC. Written informed consent was obtained from each participant.

### Apparatus and Procedure

#### Pronoun Circle Task

This experiment employed the pronoun circle task, a well-tested self-construal priming paradigm, to momentarily activate participants’ independent or interdependent self-construal. We collected two essays printed on separate sheets for priming independent or interdependent self-construal. One essay contained singular pronoun (e.g., “*I*,” “*me*,” “*my*”) to prime independent self-construal, and one essay contained plural pronouns (e.g., “*we*,” “*us*,” “*our*”) to prime interdependent self-construal. Each essay contained a total of 16 pronouns. Instructions to participants were to read each essay carefully and circle all the pronouns with a pen. To ensure task involvement, each participant had to retell the essay in several sentences and fill out the total number of pronouns in the essay. All participants in this experiment were able to retell the content of the essay well, and reported the correct number of pronouns in the essay. In the current experiment, we found that all participants showed 100% accuracy in counting the number of pronouns.

#### Ensemble Coding Task

**Figure 1:**
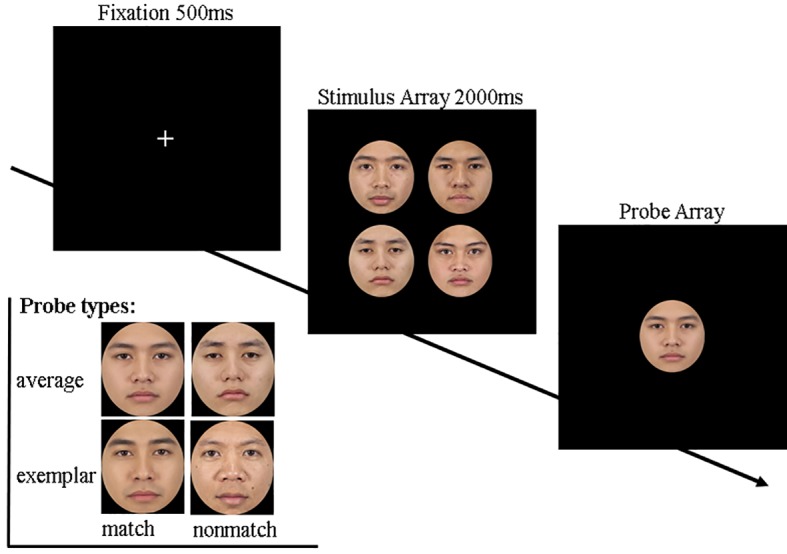
A schematic presentation of the ensemble coding task (top) and the probe types (below). Each trial began with a fixation (500 ms) and a stimulus array containing four original faces (2,000 m). Next, in the probe array, participants had to indicate whether the probe face had been “present” or “absent” in the prior array by pressing the “F” (present) or “J” (absent) key. The probe face could be either a morphed average face (set average) or a member face (exemplar), and could have been taken either from the preceding stimulus set (match condition) or from another set (nonmatch condition).

This experiment applied the implicit ensemble coding task (de Fockert and Wolfenstein, 2009) to measure ensemble perception. As shown in Figure 1, each trial began with a 500 ms central fixation cross and subsequently a set of four faces centering at fixation (2,000 ms). After that, a single probe face was presented at fixation, and participants had to judge whether the probe face had been “present” or “absent” in the preceding set. Pressing the “F” key with left index finger indicated “present,” while pressing the “J” key with the right index finger indicated “absent.” The probe face could be either a morphed average face from the preceding set (match average), a morphed average face from another set of the same gender (nonmatch average), a member face from the preceding set (match exemplar), or a member face from another set of the same gender (nonmatch exemplar). No feedback or time limit was given.

A total of 56 Asian Chinese facial images with a neutral expression chosen from the Chicago Face Database (Ma et al., 2015) was used as the material for this study. All the images were first framed within an oval shape measuring 180 px horizontally and 200 px vertically using Adobe Photoshop 6.0, to ensure that only eyes, eyebrows, nose, and mouth were visible. Following de Fockert and Wolfenstein’s (2009) procedure, we randomly created 14 face sets, each containing 4 same-gender faces, and 14 morphed average faces based on the 4 original faces of each set by Abrosoft FantaMorph 5. The procedure was written and run *via* E-prime software and presented on a 23.8-inch DELL screen. The distance between participants and the screen was about 60 cm. Each participant completed four blocks of 56 trials each (each of the 14 sets of four faces was used four times, corresponding to the four types of probe face).

### Data Analysis

Data analysis was executed using SPSS 20.0. To check the priming effect on ensemble coding of the multiple face identities, a Prime Type (independent self-construal priming vs. interdependent self-construal priming) × Testimage Type (Set Average vs. Exemplar) mixed two-way ANOVA was conducted, with the Prime Type as a between-subject variable. Endorsement scores, an unbiased index of recognition performance in ensemble coding task, were employed as the dependent variable; this was calculated by subtracting the percentage of “present” responses on the nonmatch condition from the proportion of “present” responses on the match condition for both the set average and the exemplars (Rhodes et al., 2015). For example, we computed the endorsement score of set average by subtracting the proportion of “present” responses of nonmatch average trials from the proportion of “present” responses of match average trials.

### Results

To check the manipulation of self-construal priming, we coded the self-description sentences following the methodology used in the previous studies (Gardner et al., 1999; Sui et al., 2007). Participants’ responses sentences were coded into (1) independent self-descriptions; (2) interdependent self-descriptions; or (3) unclassified responses. Consistent with previous studies (e.g., Sui et al., 2007), participants generated more independent self-descriptions (*M* = 5.50, *SD* = 2.17) than interdependent self-descriptions (*M* = 3.86, *SD* = 2.07, *p* = 0.001), given that the instructions were ask them to describe themselves. Further, the Prime Type [(independent self-construal priming vs. interdependent self-construal priming) × Self-description (independent self-description vs. interdependent self-description] mixed ANOVA, with the Prime Type as the between-subject variable, found a significant interaction (*F*(1, 50) = 34.58, *p* < 0.001, *η*^2^ = 0.409). *Post hoc* LSD tests demonstrated that more independent self-descriptions were generated in the independent self-construal priming condition (*M* = 6.96, *SD* = 2.22) than in the interdependent self-construal priming condition (*M* = 3.79, *SD* = 1.84, *p* < 0.001). In addition, significantly more interdependent self-descriptions were listed in the interdependent self-construal priming condition (*M* = 5.46, *SD* = 1.77) than in the independent self-construal priming condition (*M* = 2.50, *SD* = 1.95, *p* < 0.001). Conclusively, the manipulation check confirmed the validity of the self-construal priming task used in the current experiment.

**Figure 2:**
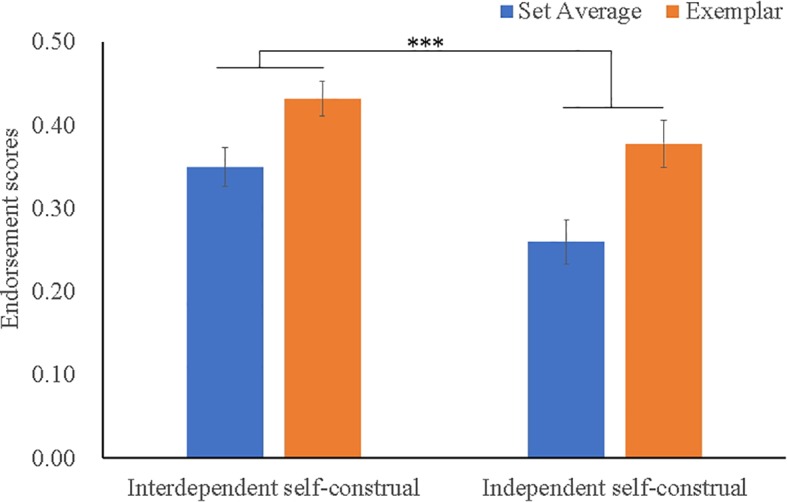
Endorsement scores as a function of Prime Type × Testimage Type in Study 1. Error bars refer to standard error of mean (SME). *** refers to the significant Testimage Type effect (*p* < 0.001).

Descriptive data of the “present” responses and the endorsement scores is presented in Table 1. The mixed ANOVA uncovered a significant effect of Prime Type (*F*(1, 50) = 5.83, *p* = 0.019, *η*^2^ = 0.104), as well as a significant Testimage Type effect (*F*(1, 50) = 26.32, *p* < 0.001, *η*^2^ = 0.345). As illustrated in Figure 2, the endorsement scores of the set average (*M* = 0.31) were less than those of the exemplars (*M* = 0.41). Additionally, participants under interdependent self-construal priming (*M* = 0.39) displayed higher endorsement scores (of both set average and exemplar) than those under independent self-construal priming (*M* = 0.32). No significant two-way interaction was found, *F*(1, 50) = 0.84, *p* = 365, *η*^2^ = 0.016.

**Table 1:** Descriptive data of experiment 1.

Self-construal priming	Proportions of “present” responses				Endorsement scores	
	Match average	Nonmatch average	Match exemplar	Nonmatch exemplar	Set average	Exemplar
Interdependent	0.63 (0.13)	0.28 (0.14)	0.70 (0.14)	0.28 (0.09)	0.35 (0.12)	0.43 (0.13)
Independent	0.57 (0.17)	0.31 (0.15)	0.64 (0.10)	0.27 (0.11)	0.26 (0.11)	0.38 (0.15)

### Discussion

The results of Experiment 1 supported our hypothesis, showing that temporarily activated interdependent self-construal (vs. independent self-construal), which featured a global-processing style and more emphasis on others than on self, enhanced the endorsement scores of the set average (0.35 vs. 0.26), as well as the exemplar (0.43 vs. 0.38). Experiment 1 indicated that interdependent self-construal boosted both individual and ensemble coding performance, which we argued make sense, as the emphasis on interdependence means the thoughts of others rather than self (Markus and Kitayama, in press). In this experiment, both exemplars and set averages were in the “others” category (non-self-face). Had there been an “own-face” in the set, then the responses to the own-face would have been affected by independent self-construal priming (Sui and Han, 2007; Sui et al., 2009). In sum, our hypothesis of Experiment 1 was confirmed, as interdependent self-construal (vs. independent self-construal priming) augmented the visual extracting of average identity of multiple faces.

## Experiment 2

### Participants

A new sample of *n* = 58 (10 males) right-handed college students from RUC was recruited to take part in this experiment, with monetary compensation. All participants had normal or corrected-to-normal vision. Participants were randomly assigned into two groups: (1) one independent self-construal priming group (*n* = 29, 6 males, age = 21.10 ± 2.47 years old); and (2) one interdependent self-construal priming group (*n* = 29, 4 males, age = 21.52 ± 2.89 years old). This experiment was approved by the Institutional Review Board of Department of Psychology, RUC. Written informed consent was obtained from each participant.

### Apparatus and Procedure

#### Pronoun Circle Task

The priming task used in this experiment was the same as that used in Experiment 1. In the current experiment, we found that all participants showed 100% accuracy in counting the number of pronouns.

#### Ensemble Coding Task

**Figure 3:**
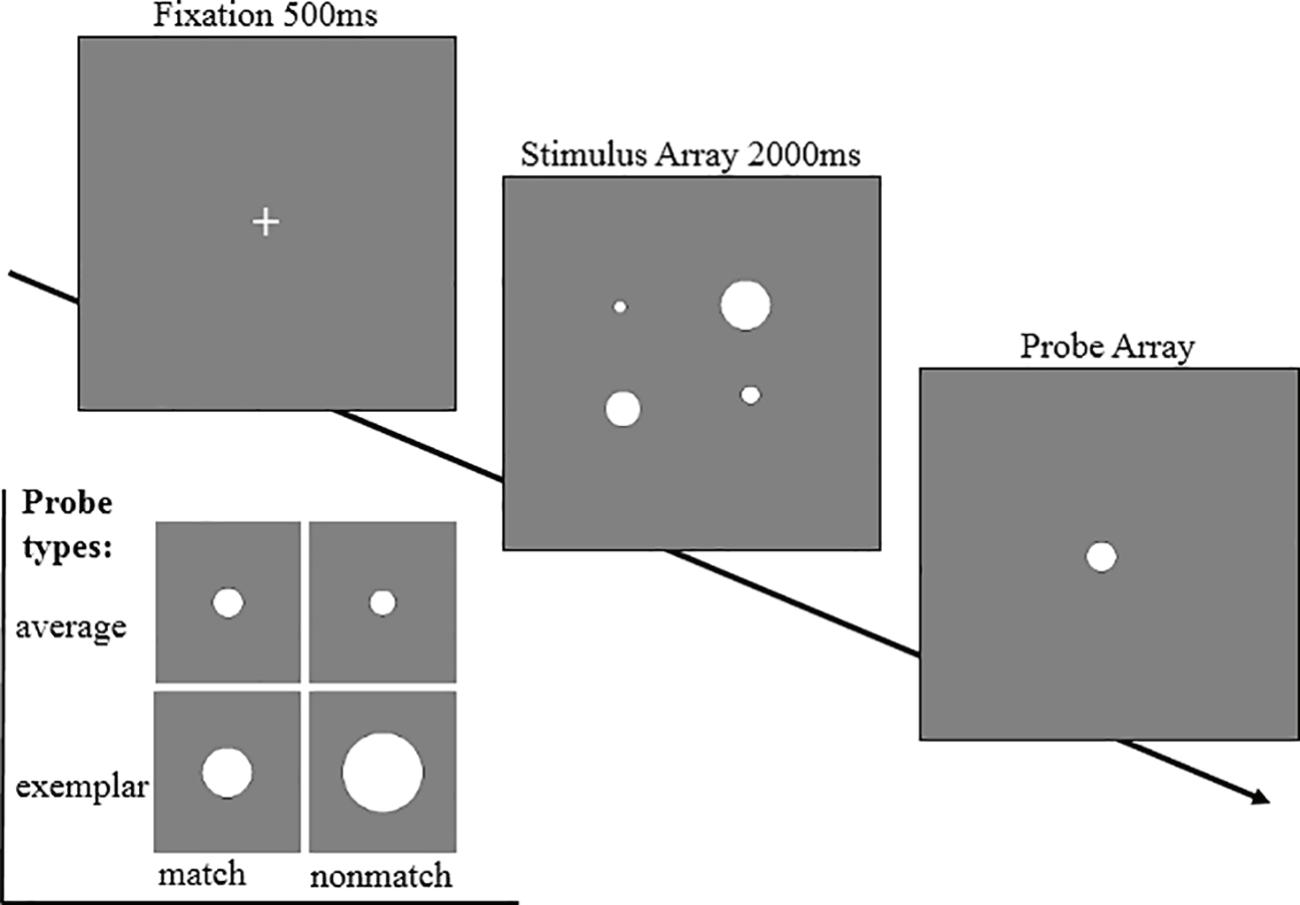
A schematic presentation of ensemble coding task and probe type used in Experiment 2. Each trial began with a fixation (500 ms) and a stimulus array containing four heterogeneous dots (2,000 m). Next, in the probe array, participants had to indicate whether the probe dot was “present” or “absent” in the prior array based on the size of the dot by pressing “F” (present) or “J” (absent) key. The probe dot could be either a dot with size equal to the mean size of the preceding set (match average), a dot with size was the mean size of another stimulus set (nonmatch average), a dot of the preceding set (match exemplar), or a dot of another set (nonmatch exemplar).

An ensemble coding task similar to Experiment 1 was employed in this experiment, with the only exception being that Experiment 2 adopted dots rather than faces as materials (Ariely, 2001). For each trial, participants were first presented with a set of four white dots of different size for 500 ms, and then shown a probe dot. Participants had to decide whether the probe dot had been “present” or “absent” in preceding set, based on the dot size (Figure 3). They were to press the “F” key with the left index finger to indicate a “present” response and press the “J” key with the right index finger to indicate an “absent” response. No feedback or time limit were given.

The dots used in this experiment were calculated by MATLAB using the procedure established by Ariely (2001). A total of 30 sets of heterogeneous dots was created, and 30 dots with their size equal to the mean size of the 30 sets of original dots were plotted and created. Each set constituted four heterogenous sizes equally spaced on a log scale. Each size was separated from the next size by a factor of 1.4(*n*). The semidiameter ranged from 0.25 to 0.4°. All dots were plotted on a 200 × 220 px gray box. Each participant completed four blocks of 60 trials each (each of the 30 sets of four faces was used eight times corresponding to the four types of probe face).

### Data Analysis

As in Study 1, data analysis in this experiment was executed using SPSS 20.0. To check the priming effect on ensemble coding of multiple face identities, a Prime Type (independent self-construal priming vs. interdependent self-construal priming) × Testimage Type (Set Average vs. Exemplar) mixed two-way ANOVA was conducted on the endorsement scores, with the Prime Type as between-subject variable.

### Results

Before entering the main analysis, a manipulation check was conducted, as in Experiment 1. We first found participants listed more independent self-descriptions (*M* = 6.66, *SD* = 2.17) than interdependent self-descriptions (*M* = 2.86, *SD* = 2.07, *p* < 0.001). The Prime Type ((independent self-construal priming vs. interdependent self-construal priming) × Self-description (independent self-description vs. interdependent self-description) mixed ANOVA uncovered a significant interaction (*F*(1, 50) = 43.84, *p* < 0.001, *η*^2^ = 0.439). *Post hoc* LSD tests demonstrated that more independent self-descriptions were generated in the independent self-construal priming condition (*M* = 7.93, *SD* = 1.60) than in the interdependent self-construal priming condition (*M* = 5.38, *SD* = 1.92, *p* < 0.001). In addition, significantly more interdependent self-descriptions were listed in the interdependent self-construal priming condition (*M* = 4.28, *SD* = 1.60) than in the independent self-construal priming condition (*M* = 1.45, *SD* = 1.43, *p* < 0.001). Conclusively, the manipulation check confirmed the validity of the self-construal priming task used in the current experiment.

**Figure 4:**
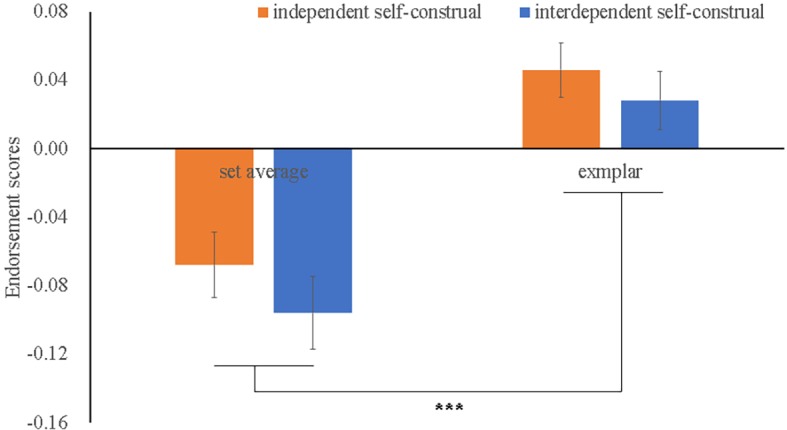
Endorsement scores as a function of Prime Type × Testimage Type in Study 2. Error bars refer to SME. *** refers to the significant Testimage Type effect (*p* < 0.001).

Descriptive data of the “present” responses and the endorsement scores is presented in Table 2. The mixed ANOVA revealed a significant Testimage Type effect (*F*(1, 56) = 37.03, *p* < 0.001, *η*^2^ = 0.398). This was driven by the fact that higher endorsement score of exemplars (*M* = 0.04) was reported than the set average (*M* = −0.08). However, as shown in Figure 4, neither the Prime Type (*F*(1, 56) = 1.79, *p* = 0.187, *η*^2^ = 0.031) nor the two-way interaction (*F*(1, 56) = 0.070, *p* = 0.792, *η*^2^ = 0.001) was significant.

**Table 2:** Descriptive data of experiment 2.

Self-construal priming	Proportions of “present” responses				Endorsement scores	
	Match average	Nonmatch average	Match exemplar	Nonmatch exemplar	Set Average	Exemplar
Interdependent	0.50 (0.14)	0.59 (0.13)	0.63 (0.14)	0.60 (0.13)	−0.10 (0.11)	0.03 (0.09)
Independent	0.45 (0.15)	0.52 (0.13)	0.61 (0.10)	0.56 (0.11)	−0.07 (0.10)	0.05 (0.09)

### Discussion

The results of Experiment 2 found that self-construal had no effect on mean size processing, which was out of line with our hypothesis that interdependent self-construal featured as global-processing would augment the ensemble perception. Given that participants were activated with independent or interdependent self-construal, respectively, based on the manipulation check, it was unlikely that the null effect of self-construal on averaging of dot size was due to the failure of the priming technique. Together with Experiment 1, the results of Experiment 2 supported the domain-specific mechanisms of ensemble perception, suggesting that different processing pathways were recruited in representing summary statistics of high-level and low-level features. However, this conclusion should be considered cautiously since the implicit ensemble coding task was first used and the endorsement scores in the current experiment were extremely low. More insights can be found in the general discussion.

## General Discussion

The present study is the first to explore the effect of independent/interdependent self-construal priming on ensemble perception, which refers to the rapid extraction of ensemble properties (e.g., mean and variation) of multiple stimuli. After being primed with either independent or interdependent self-construal through a well-validated pronoun circle task, participants had to accomplish ensemble coding of high-level features (identity, Experiment 1) and low-level features (size, Experiment 2). The results demonstrated that momentary activation of interdependent self-construal rather independent self-construal can boost the averaging of multiple face identities, but not of dot size. The current study validated the domain-specific mechanism of ensemble perception (Haberman et al., 2015), and generalized the effect of self-construal on single face recognition to multiple face recognition.

Experiment 1 supported the idea that interdependent self-construal priming, which focused more on global processing and interconnectedness with other people, can enhance the extraction of average identity. This is in line with previous studies that suggest the modulatory role self-construal plays in ensemble coding—for example, Easterners with habitual interdependent self-construal were shown to be more adept at recognizing average emotions or identity than Westerners with an independent self-construal (Im et al., 2017; Peng et al., under review). The underlying mechanism of the relationship between self-construal and ensemble perception might link the facts that the representation of ensemble properties occurs in a global-processing style (e.g., Chong et al., 2008; Peng et al., 2019), and interdependent rather independent self-construal priming can elevate the global processing (e.g., Lin and Han, 2009; Liu et al., 2015).

Self-construal affects face processing; this was confirmed in Experiment 1. As indicated by previous studies (Sui and Han, 2007; Sui et al., 2009; Ng et al., 2010), independent self-construal featured as more emphasis on self than other could improve own-face identification while interdependent self-construal featured as more stresses on social connections between people could elevate recognition of other faces (e.g., mother, friend, familiar faces). There was also evidence that self-construal modulated recognition of facial emotions (Kafetsios and Hess, 2015; Hess et al., 2016). However, whether self-construal could affect recognition of multiple faces or not, little research has been concerned with it. The one exception was Im et al. (2017), which found that Koreans did better at extracting average emotion than Americans. The task Im and colleagues adopted was not a *pure* ensemble-coding task but an avoidant task that required participants to make an avoidant choice based on the average emotion of multiple facial expressions on the both sides of the screen; therefore, other interfering factors (e.g., sensitivity to avoidant-oriented information) might have contaminated the conclusions. Thus, the current study serves as the first to tackle this issue with a *pure* ensemble-coding task, and the results extend the modulatory effect of self-construal on single-face recognition to multiple-face recognition. Given that we failed to enroll the own-face in Experiment 1, future studies are encouraged to manipulate the face materials as own-face and other-face to further explore the distinct effect of interdependent vs. independent self-construal on ensemble perception of own- and other-faces.

Experiment 2 found a dissociable results pattern from Experiment 1, showing that computing mean size of multiple dots was unaffected by self-construal priming. This was inconsistent with our assumption that ensemble perception of dot size could be modulated by the stronger tendency to global processing evoked by an interdependent self-construal priming. However, the discrepant findings of the effect of self-construal on ensemble perception of face identity and dot size were in line with the domain-specific mechanism of ensemble perception proposed by Haberman et al. (2015). In the current study, Experiment 1 found that interdependent self-construal relative to independent self-construal improved visual averaging of multiple face identities, while Experiment 2 found that self-construal did not modulate the ensemble representations of low-level feature (size). The domain-specific view suggested that although ensemble perception prevailed among different feature domains, averaging high-level (e.g., identity, emotion) and low-level (e.g., size, orientation) features recruited different operating mechanisms. Supporting this idea, prior studies (Robitaille and Harris, 2011; Neumann et al., 2017) found a discrepant set size effect on ensemble perception of face-specific stimuli (e.g., identity) and object-specific stimuli (e.g., size). Additionally, prior research has also demonstrated that ensemble perception of high-level features was reduced in clinical population (Rhodes et al., 2015; Zhang et al., 2015; Robson et al., 2018), while this was not the case for ensemble representations of low-level features (Demeyere et al., 2008; Yamanashi Leib et al., 2012). We supposed more social information and heterogeneity carried by multiple faces might be responsible for this discrepancy. To test this idea, future studies could first train participants to associate social information (e.g., self vs. other) with low-level features (e.g., Yin et al., 2019), and then explore the effect of self-construal on ensemble perception. Additionally, as suggested by Sama (2017), the different neural processing pathways recruited by high-level vs. low-level features might also partially explain their discrepant ensemble coding mechanisms, which require further study.

The current study raises questions about whether the implicit ensemble coding task is applicable for the measurement of ensemble perception of low-level features (e.g., size). To our knowledge, the ensemble coding task we used in this study was originally advanced to measure ensemble perception of face identity and has never been employed to measure the ensemble representations of low-level features before now. The data of Experiment 2 showed indiscriminate endorsement scores of the set average, with the data under two conditions being extremely low (both below zero), suggesting insensitivity to the ensemble properties. Did the outcome indicate that participants in Experiment 2 were unable to extract the mean size of multiple dots? Answers should be cautiously approached. In fact, Experiment 2 replicated the main effect of the Testimage Type as in Experiment 1, showing that endorsement scores of the exemplars were higher than the set average. This result hindered participants followed the explicit requirement of performing the member identification task. Moreover, the manipulation check of self-construal priming excluded the possibility that the results were due to the failure of priming technique. The present study could not exclude the inappropriate use of the ensemble perception task in interpreting the zero effect of self-construal on extracting the mean size. To figure out whether it could be extended to measure averaging performance of low-level features, future studies should strive for determining the underlying mechanism of the implicit ensemble coding task.

Some limitations should be acknowledged concerning this study. The first was the use of the ensemble coding task. As mentioned above, the data of Experiment 2 was extremely low, which might interfere with the results. Future studies are encouraged to explore the effect of self-construal on ensemble perception of high-level vs. low-level *via* direct measurements, such as the mean discrimination task (Ariely, 2001) and the method of adjustment (MOA, Haberman and Whitney, 2010; Sweeny et al., 2013), to confirm the current findings. Another major limitation of this study was the priming technique. In the current study, we failed to set a control condition in the self-construal priming task. As in many previous studies (Holland et al., 2004; Varnum et al., 2014; Grossmann and Jowhari, 2018), the current study was designed to compare the effect of two different self-construal primes on ensemble perception. Different result patterns under the two different self-construal primers indicated that the activated interdependent self-construal relative to independent self-construal could boost the ensemble coding of multiple-face identities. Finally, one may argue that a measure of chronic or trait self-construal orientation should be added to exclude the possible interplay effect with temporarily activated self-construal orientation. We appreciated this idea in the current study, although we suggested that it should not be an interfering factor since the participants were all from a single culture, which indicated that they embodied a similar self-construal orientation. Finally, we attributed the lack of interaction effect in Experiment 1 to fact that the faces we used were all “other” faces to participants; however, the findings were supportive but not sufficient to conclude the self-construal modulate ensemble perception of multiple faces because other confounding factors may have contributed to the between-subject differences in average identity extraction, such as face-recognition ability, which could be examined in a within-subject design (e.g., Lin and Han, 2009) in future studies.

In conclusion, this study demonstrated a dissociable effect of self-construal priming on ensemble perception of high-level vs. low-level stimuli. Specifically, interdependent self-construal relative to dependent self-construal could elevate the performance in extracting mean identity of multiple faces, while this was not the case in computing the mean size of multiple dots. The present study generalized the effect of self-construal on single-face recognition to multiple-face recognition. Furthermore, this study provided adding evidence for the domain-specific mechanism of ensemble perception and thus furthered the understanding of ensemble perception.

## Ethics Statement

This study was approved by the Institutional Review Board of Department of Psychology, Renmin University of China. Written informed consent was obtained from each participant.

## Author Contributions

SP designed this study, LZ, RX and SP collected and analyzed data, CHL, SP and PH interpreted data, SP write the draft, CHL, WC and PH revised the manuscript.

### Conflict of Interest Statement

The authors declare that the research was conducted in the absence of any commercial or financial relationships that could be construed as a potential conflict of interest.
